# Comparison of DNA methylation profiles associated with spontaneous preterm birth in placenta and cord blood

**DOI:** 10.1186/s12920-018-0466-3

**Published:** 2019-01-03

**Authors:** Xi-Meng Wang, Fu-Ying Tian, Li-Jun Fan, Chuan-Bo Xie, Zhong-Zheng Niu, Wei-Qing Chen

**Affiliations:** 1 Department of Medical Statistics and Epidemiology, Guangzhou Key Laboratory of Environmental Pollution and Health Assessment, Guangdong Provincial Key Laboratory of Food, Nutrition and Health. School of Public Health, Sun Yat-sen University, Guangzhou, Guangdong China; 2 Department of Environmental Health, Rollins School of Public Health, Emory University, Atlanta, GA USA; 3 Department of Cancer Prevention Research, State Key Laboratory of Oncology in South China, Collaborative Innovation Center for Cancer Medicine, Sun Yat-sen University Cancer Center, No 21 Qingcaigang, Jianshe Road 6, Guangzhou, 510600 Guangdong China; 4 Department of Epidemiology and Environmental Health, School of Public Health and Health Professions, State University of New York at Buffalo, 265 Farber Hall, Buffalo, NY 14214 USA; 5 Department of Information Management, Xinhua College, Sun Yat-sen University, Guangzhou, Guangdong China

**Keywords:** Epigenetics, DNA methylation, Preterm birth, EWAS

## Abstract

**Background:**

The etiology and mechanism of spontaneous preterm birth (sPTB) are still unclear. Accumulating evidence has documented that various environmental exposure scenarios may cause maternal and fetal epigenetic changes, which initiates the focus on whether epigenetics can contribute to the occurrence of sPTB. Therefore, we conducted the current study to examine and compare the DNA methylation changes associated with sPTB in placenta and cord blood.

**Methods:**

This hospital-based case-control study was carried out at three Women and Children’s hospitals in South China, where 32 spontaneous preterm births and 16 term births were recruited. Genome-wide DNA methylation profiles of the placenta and cord blood from these subjects were measured using the Illumina HumanMethylation EPIC BeadChip, and sPTB-associated differential methylated CpG sites were identified using limma regression model, after controlling for major maternal and infant confounders. Further Gene Ontology analysis was performed with *PANTHER* in order to assess different functional enrichment of the sPTB-associated genes in placenta and cord blood.

**Results:**

After controlling for potential confounding factors, one differentially methylated position (DMP) in placenta and 31 DMPs in cord blood were found significantly associated with sPTB (Bonferroni corrected *p* < 0.05). The sPTB-associated CpG sites in placenta were mapped to genes that showed higher enrichment on biological processes including biological regulation, multicellular organismal process, and especially response to stimulus, while those in cord blood were mapped to genes that had higher enrichment on biological processes concerning cellular process, localization, and particularly metabolic process.

**Conclusion:**

Findings of this study indicated that DNA methylation alteration in both placenta and cord blood are associated with sPTB, yet the DNA methylation modification patterns may appear differently in placenta and cord blood.

**Electronic supplementary material:**

The online version of this article (10.1186/s12920-018-0466-3) contains supplementary material, which is available to authorized users.

## Background

Preterm birth (PTB) is defined as birth with earlier than 37 weeks’ gestational age [1], it occurs in 11% of live births [2], and 65–70% PTB happens spontaneously. PTB is considered as not only the leading cause of neonatal death [3], but also the prominent risk factor for subsequent onset of morbidities including infections in newborns [4], severe neural system damages and various cognition impairments in childhood [5], as well as hypertension, diabetes and coronary heart disease in adulthood [6, 7].

The causes of PTB have been speculated with a series of adverse maternal and environmental factors, namely maternal active/passive smoking [8–10], mental stress [11, 12], intrauterine infection [13], malnutrition [14, 15], and environmental pollutants [11, 16, 17]. These accumulating evidence have implied that PTB should be considered as a syndrome resulting from a complex combination of various causes and pathological processes [18, 19].

Recently, expanding studies have focused on associations between PTB and abnormal DNA methylation variation in maternal tissues, placenta or cord blood. For instance, DNA methylation changes of *PTGES*, *PTGIS*, *PTGDR2*, *PGR*, and *PTGER2* in maternal myometrium and cervical swabs samples were found to be associated with PTB [20, 21]. The promoter region of *CYTIP* and *LINC00114* were found hypomethylated in preterm maternal peripheral blood [22], but no significant DNA methylation changes in maternal blood were identified in Parets’s study [23]. Moreover, DNA methylation changes of *UCN* [24], *OXTR* [24, 25], *RUNX3* [26] and *VEGF* [27] were observed in PTB placenta. Regarding cord blood, Schroeder et al. [28] found that DNA methylation variations of 39 genes (including *AVP*, *OXT,* and *CRHBP)* were associated with gestational age (GA). Fernando’s study [29] identified 1151 GA-related differentially methylated positions (DMPs) in numerous genes in cord blood (including *IGF2BP1*, *OTOF*, *ATP2B2*, *NCOR2*, *PYCR2*, and *RARA*). Parets et al. [30] discovered 29 PTB-associated and 9637 GA-associated DMPs in cord blood, and some of them (e.g., *DNMT1*, *DNMT3A*, *DNMT3B,* and *TET1)* were related to methylation regulation.

Unfortunately, most of the previous studies only focused on specific genes in one tissue (maternal tissue, placenta or cord blood). Up to now, five studies were conducted from a genome-wide perspective that used Illumina 450 K BeadChip or more advanced measurements [22, 23, 29–32], only Parets et al. and Hong et al. investigated DNA methylation status in both maternal and cord blood samples. Thus, there still lacks studies that could elaborate varied DNA methylation alternation among different tissues. Additionally, these previous studies were all conducted among Caucasian or African American populations but no Asian or Chinese populations, and some of them did not exclude all the CpG sites that might be associated with genetic variation among different races. Due to the potential influence on DNA methylation of ethnicity [33, 34], the existing studies may not provide results that can be ideally extrapolated to the Chinese population.

Hence, we conducted a case-control study to investigate the association of genome-wide DNA methylation profile with spontaneous preterm birth (sPTB) in both placenta and cord blood concurrently in a Chinese population and to identify different DNA methylation alterations in these two respective tissues.

## Methods

### Study design and participants

This is a hospital-based case-control study, and the participants of this study were recruited from September 2009 to March 2011 at three Women and Children’s Hospitals located in Shenzhen, Foshan, and Guangzhou of Guangdong Province, China. A total of 48 singleton natural-labored mother-infant pairs were enrolled in the current study, consisting of 32 preterm birth newborns with either low or normal birth weight and 16 term birth (gestational age of 37–42 weeks) newborns with normal birthweight. In this study, sPTB was defined as vaginally delivered spontaneous preterm births with a gestational age of 32–36 weeks, with or without premature rupture of membranes (PPROM). Subjects of the case group were randomly selected from all sPTB subjects with complete information, and the controls were individually matched with the 16 subjects with normal birthweight in the sPTB case group for maternal age (± 5 years), history of preterm birth and parity. Participants with the following characteristics were excluded: 1) mothers were with any of the following diseases: hypertension, diabetes, gestational diabetes mellitus (GDM), hypertensive disorders during pregnancy (HDP), hyper- or hypo- thyroidism, anemia and tuberculosis; and 2) infants had malformations at delivery or hereditary diseases (e.g. thalassemia, G6PD deficiency).

### Data collection

Maternal socio-demographic characteristics, medical and reproductive history were collected via clinic interviews after delivery, and pregnancy information (e.g., pregnancy complications, offspring biometric measures at birth) were extracted from medical records by trained study staff, the questionnaire used in the interviews had been employed in our previous works [35–37]. Details of delivery were measured and recorded by midwives, including birth weight (measured to the nearest 5 g), birth length, head circumference, as well as placental diameters and thickness. The last menstrual period (LMP) was self-reported by mothers at their first prenatal care visit (at 8–10th week of gestation) and was confirmed by early ultrasound assessment at the gestational age of less than 20 full weeks. Whereas self-reported LMP was unavailable, ultrasound estimated LMP based on the crown-rump length in early pregnancy was used instead [38]. We then calculated gestational age as the interval between LMP and delivery.

### Biological sample collection

A volume of 5 ml of umbilical venous blood was drawn by midwives with EDTA tubes as soon as a newborn was delivered. Immediately after the placenta was delivered, 1cm^3^ placenta tissue was collected without the membrane from the middle points on the radius of maternal side placenta (in order to avoid calcification points and blood clots). The placenta sample was cleaned with cold saline to remove blood in the placenta vessel. All of the cord blood and placenta samples were stored at − 80 °C.

### DNA methylation measurement and quality control

Cord blood DNA was extracted with TIANamp Genomic DNA Kit (TIANGEN Biotech, Beijing, China). Purified DNA was quantified and qualified using NanoDrop 2000 (Thermo Scientific™, San Jose, USA) and DNA gel electrophoresis (Major science, Saratoga, USA). Placenta tissue DNA was extracted with DNeasy Blood & Tissue Kit (QIAGEN, Valencia, USA). DNA extracted from cord blood and placenta tissue were bisulfite-converted using EZ DNA Methylation Kit (Zymo Research, Irvine, USA), and then amplified, fragmented and hybridized on the Infinium Methylation EPIC BeadChip (Illumina, San Diego, USA) following the manufacturer’s protocols. This latest version of methylation beadchip is able to interrogate over 850,000 methylation sites quantitatively across the genome at a single-nucleotide resolution, and it covers over 90% of the contents on the traditional 450 K BeadChip and additional 413,743 sites [39]. The samples were randomly allocated across 12 EPIC array chips which were scanned with Illumina iScan, and the original data were read with the *minfi* package [40] in the R software [41]. The *funtooNorm* package [42], which was capable of processing data from multiple tissues, was applied to calculate β-values [β = M/ (U + M), where M stood for methylated bead and U stood for unmethylated bead) and perform data normalization, as well as to correct background and dye-bias.

In this study, we excluded the following types of probes: 1) 5926 sites due to missing data; 2) 19,681 probes targeting sites on sex chromosome, 2932 non-CpG targeting probes and another 43,255 probes with cross-hybridization [39]; 3) 59 built-in explicit SNP probes, 9156 probes with genetic variants overlapping targeted CpG sites, 323 probes with genetic variants overlapping single base extension sites for Infinium Type I probes, and 87,642 probes with genetic variants overlapping the rest of the EPIC probe [39]; 4) 69,551 probes with extreme mean methylation level (mean β-value < 0.05 or > 0.95); and 5) 99,217 probes from the placenta samples and 112,239 probes from the cord blood samples due to low variation (β range < 0.05) [43]. A final number of 551,326 placenta probes and 538,304 cord blood probes were included in the statistical analysis. In addition, since all samples were randomly allocated on 12 chips in the same batch, *ComBat* procedure of the *sva* package [44] was employed to adjust for potential chip effect. With the fully processed dataset, the *log*_*2*_ ratio of *β*-values was calculated and denoted as M-values [M = *log*_*2*_*β* - *log*_*2*_ (1-*β*)), which was used in statistical analyses along with β-values; meanwhile, β-values was used for interpretation of the results.

### Statistical analysis

#### Description of participants’ demographic and clinical characteristics

Mean and standard deviation (SD) were used for continuous variables with normal distribution, and proportions were used for categorical variables. Student’s t-test and chi-squared test were used when appropriate to compare the differences of demographic and clinical characteristics between sPTB cases and term controls.

#### Estimation of cell type proportions of placenta and cord blood

Due to lack of acknowledged reference panel, cell type proportions of placenta were estimated with Reference-Free Adjustment for Cell-Type composition (ReFACTor), which was based on a variant of principal component analysis [45]. Cord blood cell type proportions were estimated using the 450 K reference panel by Bakulski et al. [46, 47] with an adjusted approach [48].

#### Identification of differential methylated positions (DMPs) associated with sPTB

Differential methylation analysis on sPTB and term birth controls was performed using the *limma* package [49]. Bonferroni correction for multiple testing was further applied, and a two-sided *p*-value less than 0.05 was considered statistically significant. Linear models were fitted to analyze the association of placenta and cord blood methylation with sPTB, adjusting for potential confounders including maternal age, newborn gender, maternal education level, pre-pregnancy BMI, and when appropriate, either the first 5 principal components obtained from the ReFACTor function or the estimated cell-type proportions (CD8 T cells, CD4 T cells, NK cells, B cells, monocyte, and nucleated red blood cells).

#### Comparison of sPTB-associated CpG sites between placenta and cord blood

First, partial correlation was performed to investigate if DNA methylation level of the sPTB-associated CpG sites in placenta and cord blood were correlated, controlling for sPTB status, maternal age, maternal education level, maternal pre-pregnancy BMI and newborns’ gender. Second, gene annotations of sPTB-associated CpG sites in placenta and cord blood were performed basing on the Infinium HumanMethylation EPIC manifest (version 1.0) and the UCSC annotation database [50]. For the CpG sites enrolled in Gene Ontology (GO) analysis, we set the thresholds to FDR < 0.05 with over 20% β-value difference between sPTB and control for the cord blood, and *p*-value < 1.0E-03 with over 20% β-value difference between sPTB and control for the placenta. Then, GO-biological process enrichment was performed with PANTHER 12.0 [51] to interpret the function of genes covering the sPTB-associated CpG sites, and to compare the functional differences of aberrantly methylated genes in placenta and cord blood.

## Results

### Subject description

The demographic and clinical characteristics of participants are presented in Table 1. sPTB newborns had significantly higher pre-pregnancy BMI in their mothers, and had significantly lower weight, length and head circumference at birth. Furthermore, no significant differences were observed concerning maternal age, education level, alcohol use, placenta surface and thickness, and newborn gender. None of the mothers actively smoked cigarettes during pregnancy in this study, and the proportion of passive smoking showed no significant difference between sPTB and control.

**Table 1:** Demographic and clinical characteristics of study participants

	sPTB (*N* = 32)	Term Birth (*N* = 16)	*p*-value
Mother			
Maternal age (years), mean (SD)	27.16 (4.14)	27.19 (3.90)	0.98
Pre-pregnancy BMI, mean (SD)	20.06 (2.22)	18.80 (1.13)	0.04
College or above education, N (%)	17 (53.10)	7 (43.80)	0.54
Family income > ¥3000/month, N (%)	17 (53.10)	9 (56.30)	0.84
Primiparity, N (%)	28 (87.50)	13 (81.30)	0.67
Passive smoking, N (%)	22 (68.80)	8 (50.00)	0.21
Alcohol use, N (%)	1 (3.10)	2 (12.50)	0.25
Placenta			
Placental surface area (cm^2^), mean (SD)^a^	260.41 (59.17)	286.43 (41.91)	0.12
Placenta thickness (cm), mean (SD)	2.19 (0.47)	2.34 (0.47)	0.29
Newborn			
Gestational age (weeks), mean (SD)	35.09 (1.35)	39.63 (1.09)	< 0.001
Gender (male), N (%)	59.40% (19/32)	56.30% (9/16)	0.84
Birth weight (gram), mean (SD)	2436.53 (461.48)	2999.38 (268.28)	< 0.001
Birth length (cm), mean (SD)	46.50 (2.70)	49.44 (1.63)	< 0.001
Head circumference (cm), mean (SD)	31.13 (1.31)	32.38 (1.31)	0.003

^a^ The placental surface area was calculated using the formula for the area of ellipse: major axis × minor axis × π/4

### DNA methylation associated with sPTB in placenta and cord blood

**Fig. 1:**
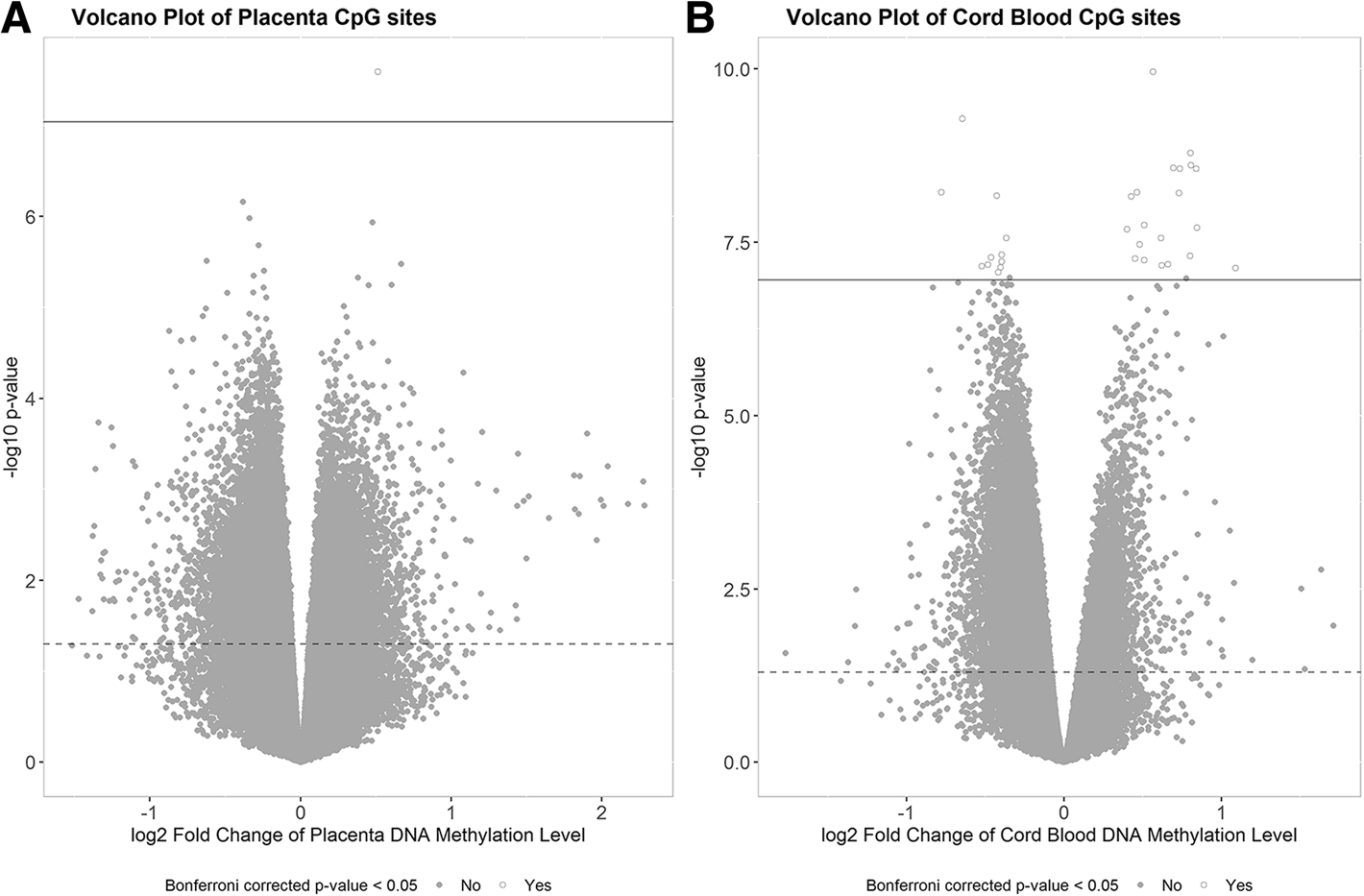
Volvano plot of the association between placenta/cord blood DNA methylation and sPTB. **a** Volcano plot of the differentially methylated positions associated with sPTB in placenta. **b** Volcano plot of the differentially methylated positions associated with sPTB in cord blood. The x axis represents log2 transformed fold changes of DNA methylation level (M-values) between sPTB and term birth. The y axis represents log_2_transformed *p*-values of the associations between each differentially methylated position and sPTB. The dashed horizontal lines represent *p*-value = 0.05, the solid horizontal lines represent Bonferroni corrected *p*-value = 0.05

Figure 1 shows the volcano plot of the association between placenta and cord blood DNA methylation and sPTB. The CpG sites in cord blood were more tended to be hypomethylated in the sPTB group **(**Fig. 1**-**b), and those in placenta did not show significant tendency (Fig. 1**-**a).

**Fig. 2:**
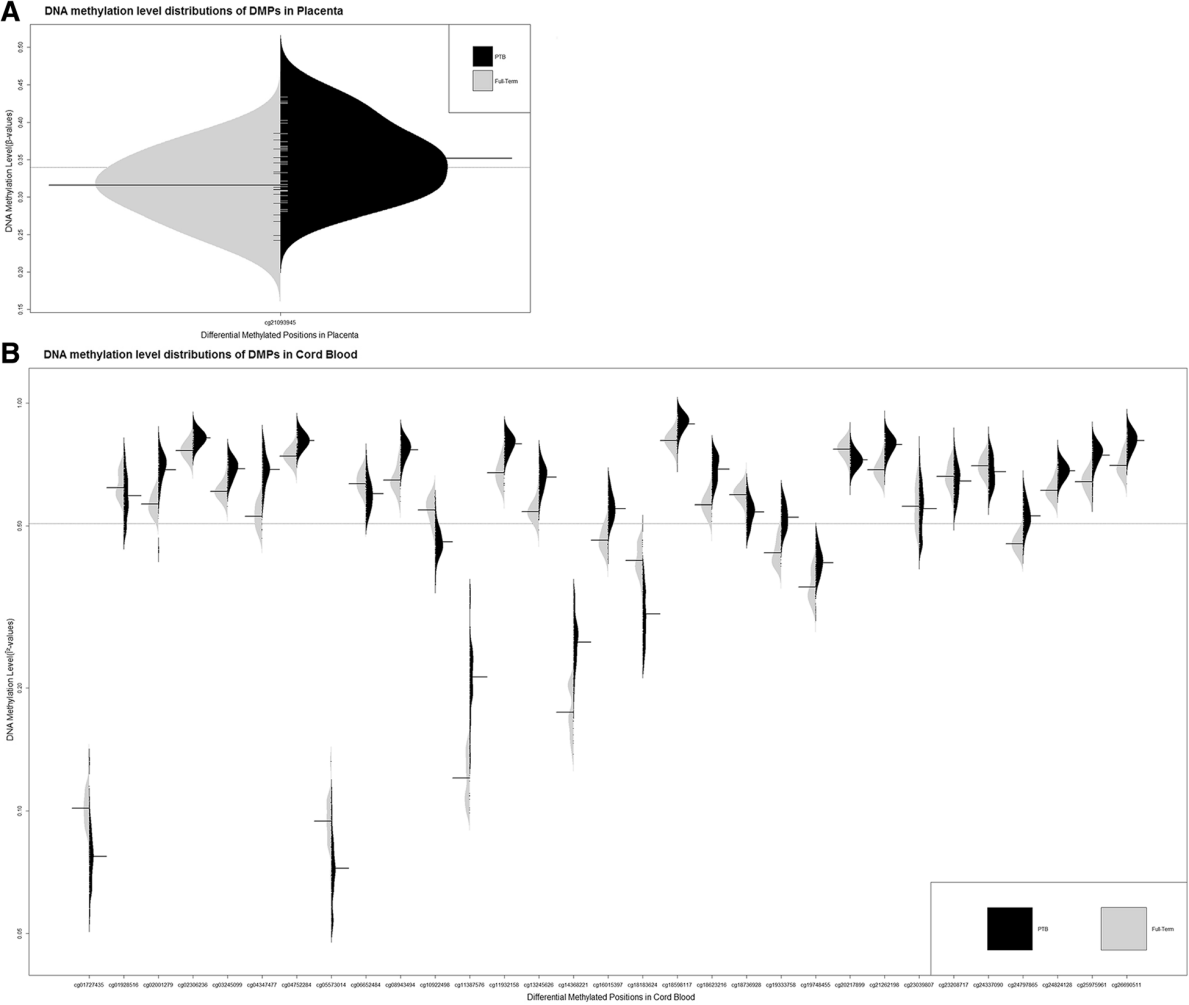
DNA methylation level distribution of significant DMPs in placenta and cord blood. **a** DNA methylation level distribution of significant DMPs in placenta. **b** DNA methylation level distribution of all significant DMPs in cord blood. The black horizontal lines in the beanplots represent the mean β-values of DMPs in the case and control groups. The dashed horizontal lines represent the mean β-values of all DMPs in each graph

After adjusting for maternal age, newborn gender, maternal education level, pre-pregnancy BMI and first five principal components obtained from the ReFACTor function, the linear model found that 43,638 CpG sites in placenta were associated with sPTB (*p* < 0.05). Only one site (cg21093945) remained significant after Bonferroni correction for multiple testing (*p* < 9.07 × 10^− 8^), which was hypermethylated in sPTB placenta. (Table 2 and Fig. 2).

**Table 2:** Significant differential methylated sites in placenta and cord blood

CpG sites	ALL Mean(SD)	sPTB Mean(SD)	FT-NBW Mean(SD)	*p*-value^*a*^	Association coefficient (SD)^b^	Chrom-osome	Position	Relation to CpG island	Gene	Region	450 K Loci
Placenta											
cg21093945	0.340 (0.05)	0.352 (0.05)	0.316 (0.04)	2.57E-08	0.08 (0.03)	chr1	56,107,046	OpenSea	RP11-466 L17.1	5’UTR	NO
Cord Blood											
cg03245099	0.663 (0.05)	0.691 (0.03)	0.607 (0.03)	5.41E-08	0.07 (0.03)	chr1	28,201,862	Shelf	THEMIS2	Body	NO
cg24824128	0.658 (0.04)	0.682 (0.03)	0.610 (0.03)	6.90E-09	0.07 (0.02)	chr1	28,202,085	Shelf	THEMIS2	Body	NO
cg20217899	0.741 (0.03)	0.726 (0.03)	0.771 (0.03)	4.79E-08	−0.05 (0.02)	chr2	218,890,650	OpenSea	/	/	NO
cg18736928	0.560 (0.04)	0.542 (0.04)	0.597 (0.03)	6.01E-08	−0.07 (0.03)	chr2	219,303,519	OpenSea	VIL1	Body	NO
cg23039807	0.559 (0.08)	0.557 (0.08)	0.562 (0.07)	8.58E-08	−0.07 (0.03)	chr2	240,425,897	OpenSea	/	/	YES
cg18623216	0.648 (0.08)	0.690 (0.05)	0.564 (0.03)	2.73E-08	0.10 (0.04)	chr3	155,421,970	OpenSea	PLCH1	5’UTR	YES
cg26690511	0.774 (0.06)	0.809 (0.04)	0.703 (0.04)	2.71E-09	0.09 (0.03)	chr3	155,422,103	OpenSea	PLCH1	TSS200	YES
cg11932158	0.755 (0.07)	0.795 (0.04)	0.675 (0.04)	1.63E-09	0.11 (0.04)	chr3	155,422,129	OpenSea	PLCH1	TSS200	YES
cg21262198	0.756 (0.06)	0.791 (0.03)	0.686 (0.03)	2.66E-09	0.09 (0.03)	chr3	155,422,159	OpenSea	PLCH1	TSS200	NO
cg13245626	0.620 (0.07)	0.659 (0.05)	0.543 (0.03)	6.83E-08	0.10 (0.04)	chr4	75,558,301	OpenSea	/	/	NO
cg05573014	0.081 (0.02)	0.074 (0.01)	0.095 (0.01)	5.16E-10	−0.03 (0.01)	chr6	150,255,468	OpenSea	RP11-244 K5.4	TSS200	NO
cg23208717	0.651 (0.06)	0.646 (0.06)	0.661 (0.05)	2.75E-08	−0.06 (0.02)	chr7	150,082,688	OpenSea	ZNF775	5’UTR	YES
cg25975961	0.711 (0.07)	0.746 (0.05)	0.641 (0.03)	6.15E-09	0.11 (0.04)	chr7	150,600,818	OpenSea	/	/	NO
cg14368221	0.233 (0.05)	0.262 (0.04)	0.176 (0.02)	6.61E-08	0.08 (0.04)	chr9	18,260,702	OpenSea	/	/	NO
cg11387576	0.186 (0.06)	0.219 (0.05)	0.121 (0.02)	1.96E-08	0.08 (0.04)	chr9	18,260,848	OpenSea	/	/	NO
cg06652484	0.612 (0.05)	0.601 (0.05)	0.635 (0.04)	7.25E-08	−0.07 (0.03)	chr9	129,242,893	Shore	FAM125B	TSS200	NO
cg24337090	0.687 (0.05)	0.680 (0.06)	0.702 (0.03)	6.74E-09	−0.06 (0.02)	chr11	11,578,126	OpenSea	GALNT18	Body	NO
cg08943494	0.728 (0.07)	0.769 (0.04)	0.648 (0.04)	2.43E-09	0.11 (0.04)	chr11	36,422,615	OpenSea	PRR5L	5’UTR	YES
cg19333758	0.494 (0.06)	0.526 (0.04)	0.430 (0.03)	5.74E-08	0.09 (0.04)	chr12	48,135,549	OpenSea	RAPGEF3	Body	NO
cg04347477	0.636 (0.09)	0.689 (0.06)	0.529 (0.04)	2.75E-09	0.14 (0.05)	chr12	125,002,007	Island	NCOR2	5’UTR	YES
cg02306236	0.803 (0.04)	0.821 (0.02)	0.765 (0.03)	1.78E-08	0.06 (0.02)	chr12	123,570,372	OpenSea	PITPNM2	5’UTR	NO
cg01928516	0.606 (0.07)	0.597 (0.08)	0.622 (0.06)	5.27E-08	−0.08 (0.03)	chr17	2,208,377	Shore	SMG6	TSS1500	NO
cg18183624	0.343 (0.07)	0.309 (0.06)	0.412 (0.04)	5.99E-09	−0.12 (0.05)	chr17	47,076,904	Shore	IGF2BP1	Body	YES
cg19748455	0.390 (0.04)	0.408 (0.04)	0.355 (0.03)	2.04E-08	0.07 (0.02)	chr17	76,274,856	OpenSea	LOC100996291	TSS1500	NO
cg10922498	0.489 (0.06)	0.459 (0.04)	0.549 (0.04)	6.64E-08	−0.08 (0.03)	chr18	70,900,410	OpenSea	LOC400655	Body	NO
cg02001279	0.648 (0.08)	0.690 (0.07)	0.566 (0.03)	5.02E-08	0.13 (0.05)	chr19	940,967	Island	ARID3A	Body	YES
cg18598117	0.864 (0.06)	0.890 (0.05)	0.810 (0.03)	7.49E-08	0.09 (0.04)	chr19	941,126	Island	ARID3A	Body	YES
cg01727435	0.086 (0.02)	0.079 (0.02)	0.102 (0.01)	7.04E-08	−0.03 (0.01)	chr20	3,767,756	Shore	CDC25B	5’UTR	NO
cg24797865	0.505 (0.05)	0.531 (0.04)	0.452 (0.02)	5.99E-09	0.08 (0.03)	chr21	46,331,470	OpenSea	ITGB2	5’UTR	NO
cg16015397	0.523 (0.06)	0.553 (0.05)	0.462 (0.03)	1.10E-10	0.10 (0.03)	chr21	46,331,472	OpenSea	ITGB2	5’UTR	NO
cg04752284	0.787 (0.04)	0.810 (0.03)	0.741 (0.02)	3.39E-08	0.06 (0.02)	chr21	46,386,748	Shore	FAM207A	Body	NO

^a^*p* values were generated based on M-values of the CpG sites in the limma regression model

^b^ The association coefficients were generated based on β-values of the CpG sites within the same model

After adjusting for maternal age, newborn gender, maternal education level, pre-pregnancy BMI and estimated cell-type proportions (CD8 T cells, CD4 T cells, NK cells, B cells, monocyte and nucleated red blood cells), the linear model identified that 72,711 CpG sites in cord blood were associated with sPTB (*p* < 0.05), and 31 of them remained significant after correcting for multiple testing (*p* < 9.28 × 10^− 8^). Among these 31 sites, 20 were hypermethylated and 11 were hypomethylated only in the cord blood of sPTB cases. More details are presented in Table 2 and Fig. 2. Furthermore, sensitivity analysis was performed for both placenta and cord blood models (Additional file 1: Figure S1).

**Fig. 3:**
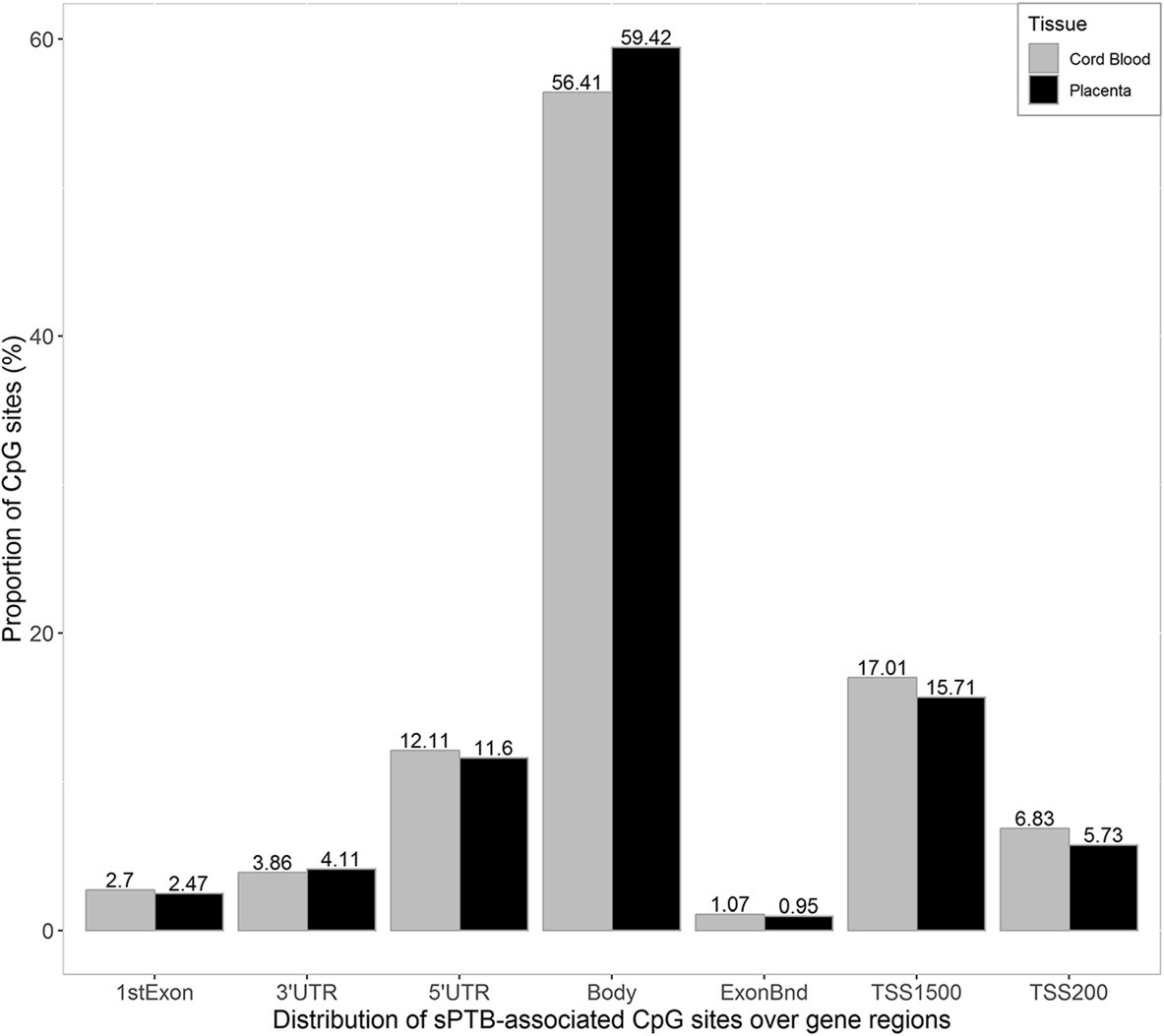
Illustration of the distribution of sPTB-associated CpG sites over gene regions in placenta and cord blood. The TSS200: region 200 base pairs within the transcription start site (TSS); TSS: region 1500 base pairs within the TSS excluding the TSS 200 region; UTR: untranslated region as present in the mRNA molecule, specifically, 5′ of the transcription start site (5’ UTR) and 3′ of the termination signal (3’ UTR); Body: coding and non-coding regions from the TSS until the termination codon

**Fig. 4:**
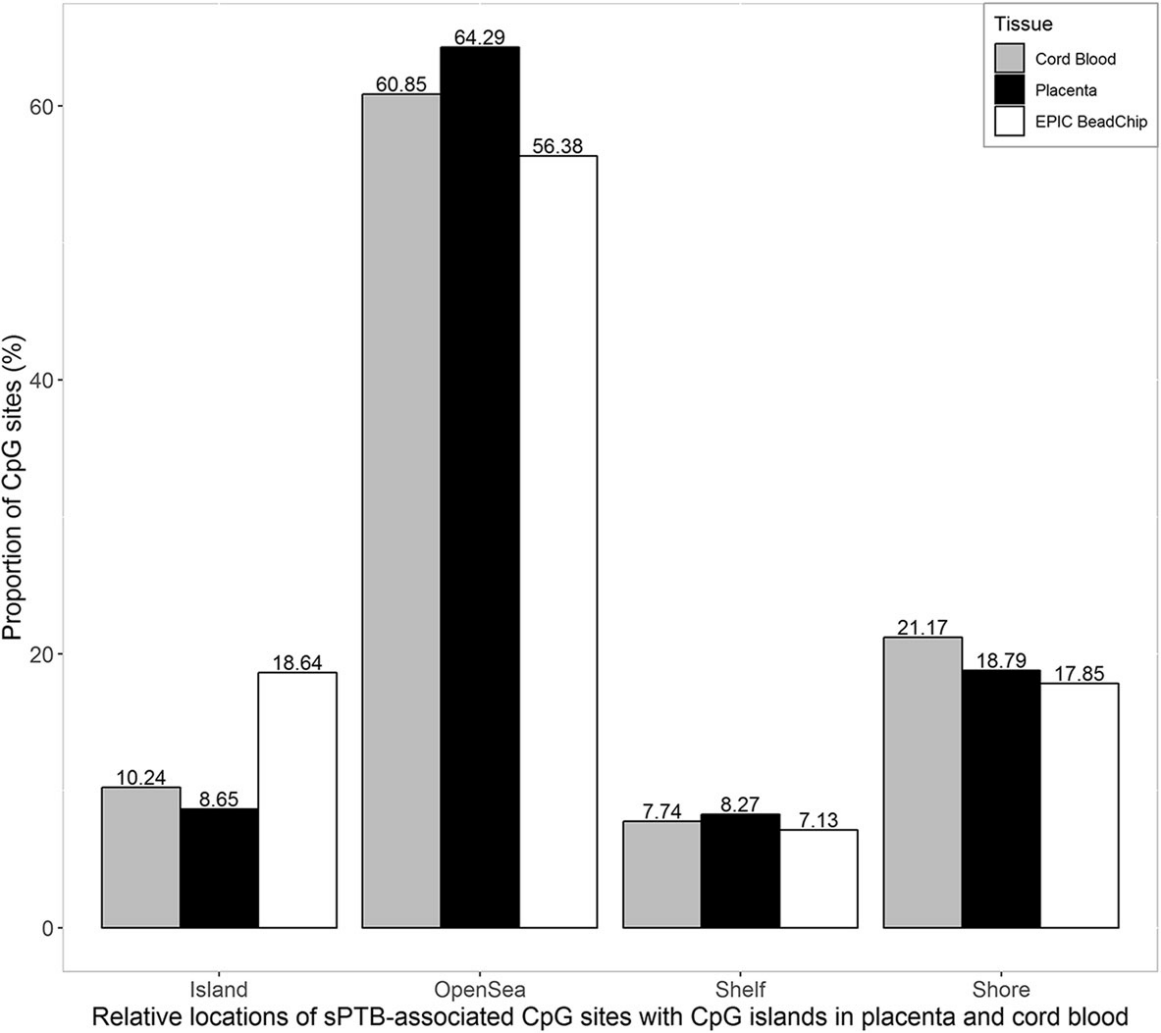
Relative locations of sPTB-associated CpG sites with CpG islands in placenta and cord blood. ‘Shore’ represent the CpG island shore regions. ‘Shelf’ represents the CpG island shelf regions. ‘Opensea’ represents the opensea regions of the genome

Basing on the Infinium HumanMethylation EPIC manifest and the UCSC annotation database [50], gene annotations were performed on sPTB-associated CpG (*p* < 0.05). A total of 29,663 CpG sites in placenta and 50,658 CpG sites in cord blood were annotated to specific genes. Figure 3 presents the distribution of CpG sites over gene regions in placenta and cord blood. For the CpG sites in placenta, 59.42% (*n* = 17,626) sites located on gene-body, 4.11% (*n* = 1220) sites were on 3’ UTR, and 11.6% (*n* = 3442) sites were on 5’UTR. Regarding the CpG sites in cord blood, 56.41% (*n* = 28,577) sites were located on gene-body, 3.86% (*n* = 1956) sites on 3’ UTR, and 12.11% (*n* = 6135) sites on 5’UTR. Figure 4 presents the relative locations of the CpG sites subset with CpG islands in placenta and cord blood, as well as all CpG sites of the EPIC BeadChip. Among the sPTB-associated CpG sites in placenta, 8.65% located on CpG islands, and 64.29% were on the open sea regions. Meanwhile, 10.24% of the cord blood CpG sites located on CpG islands, and 60.85% were on the open sea regions.

### Comparing differences of sPTB-associated DNA methylation variations in placenta and cord blood

**Fig. 5:**
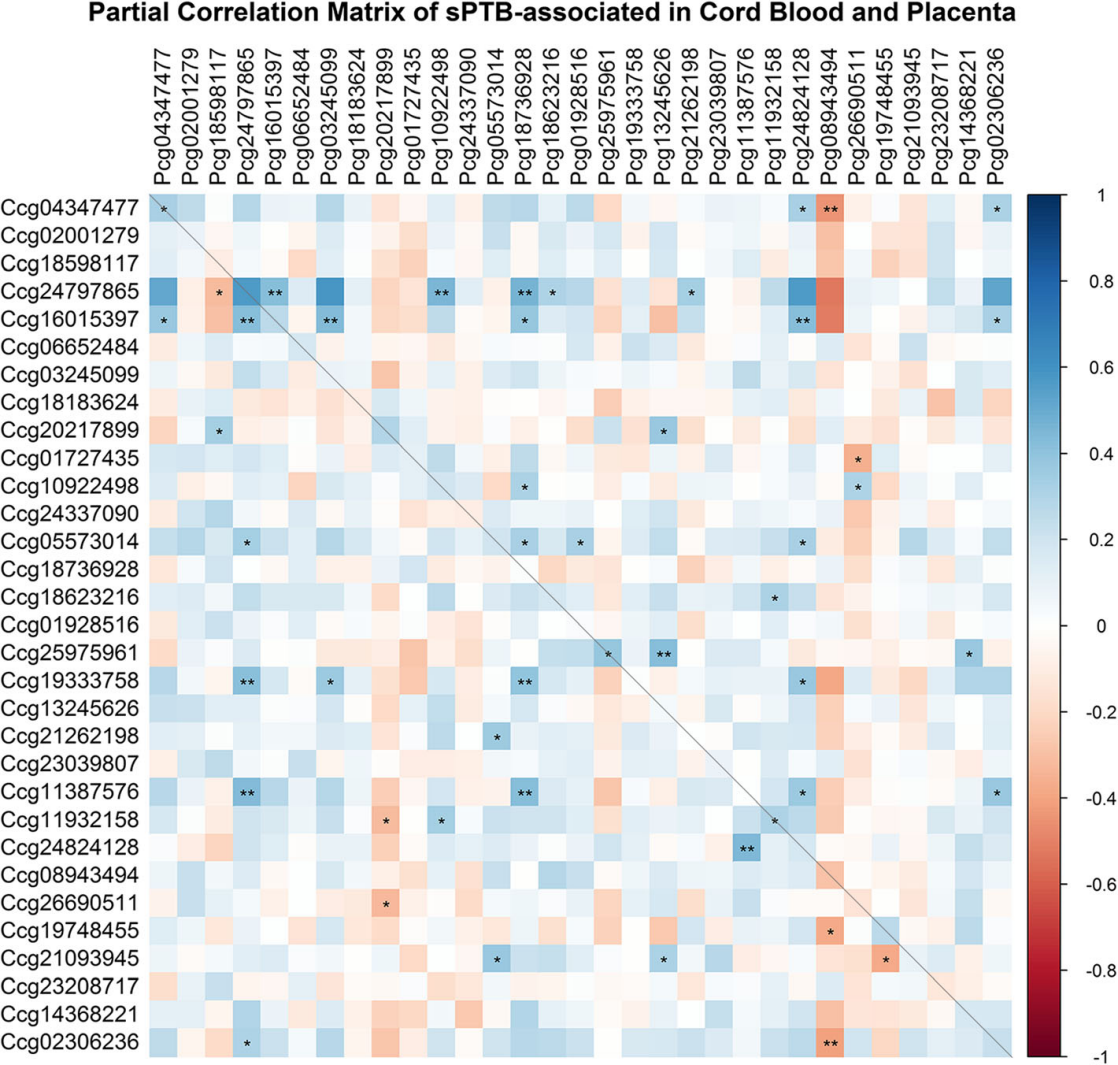
Partial correlation matrix of sPTB-associated CpG sites in Cord Blood and Placenta. Partial correlation matrix between DNA methylation (DNAm) level of combined 32 CpG sites in cord blood and DNAm of those CpG sites in placenta, controlling for status of sPTB, maternal age, maternal education level, maternal pre-pregnancy BMI and newborns’ gender. The x axis represents the CpG sites in placenta. The y axis represents the CpG sites in cord blood. The dashed line highlights the correlation between DNAm of CpG sites in one tissue with the same particular CpG site in the other tissue. * *p*-value < 0.05, ** *p*-value < 0.001

After controlling for sPTB status, maternal age, maternal education level, maternal pre-pregnancy BMI and newborns’ gender, partial correlation analysis revealed that only the DNA methylation level of cg04347477 and cg25975961 showed significant positive correlations between placenta and cord blood (Fig. 5).

**Fig. 6:**
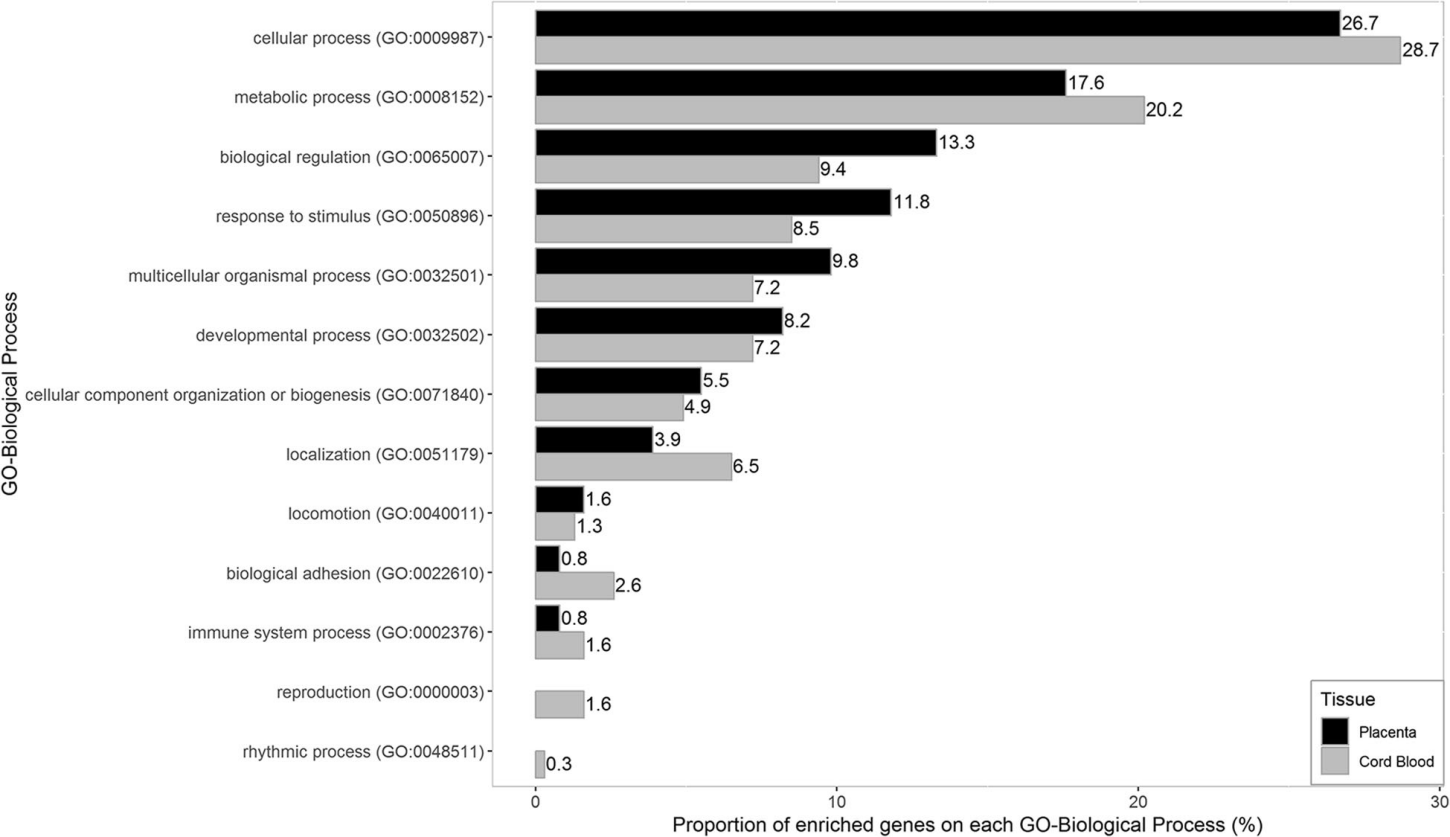
Biological process enrichment of genes covering sPTB-associated CpG sites in placenta and cord blood

A total of 841 CpG sites in placenta (*p*-value < 1.0E-03 with over 20% β-value difference between case and control) and 946 CpG sites in cord blood sites (FDR < 0.05 with over 20% β-value difference between case and control) were included for biological process enrichment. The sPTB-associated CpG sites in placenta were mapped to genes that principally enriched on 11 biological processes, and those in cord blood were mapped to genes that enriched on 13 biological processes. However, different tendency of biological process enrichment was found between placenta and cord blood: genes covering sPTB-associated CpG sites in placenta showed higher enrichment on biological processes including biological regulation, multicellular organismal process, and especially response to stimulus, while those in cord blood had higher enrichment on biological processes concerning cellular process, localization, and particularly metabolic process (Fig. 6).

## Discussion

The current study first applied the Infinium MethylationEPIC BeadChip to investigate the association of placenta and cord blood DNA methylation profiles with sPTB in the homogenous Chinese population. We found that 43,638 CpG sites in placenta were associated with sPTB, and only one CpG site remained significant after further Bonferroni correction for multiple testing. Likewise, 72,711 CpG sites in cord blood were identified to be associated with sPTB independently, and 31 of them passed Bonferroni correction for multiple testing. Besides, GO-biological process enrichment observed in our study suggested that a higher proportion of genes covering the identified sPTB-associated CpG sites in placenta were enriched on biological regulation, multicellular organismal process and response to stimulus, while higher proportions of the genes covering the identified sPTB-associated CpG sites in cord blood were instead enriched on cellular process, localization and metabolic process.

### DNA methylation associated with sPTB in placenta and cord blood

Recently, Kim et al. [24] employed high-throughput technique and identified 65 CpG sites with altered DNA methylation in PTB placenta, and the CpG sites were annotated with 61 genes such as *TOB1*, *PNPLA3*, *ZNF671*, *DAB2IP*, *MFNG*, *UCN, EXOC3L2, SLC44A2, FBXL19-AS1, DLGAP5, SLC30A3, CHFR, C11orf1, SLC24A4,* and *PI4KB*. Moreover, several studies have documented the association between PTB and DNA methylation in specific genes like *RUNX3* [26], *VEGF* [27], *KDR* [27] and *OXTR* [25] in the placenta. In the present study, we identified 43, 638 CpG sites associated with PTB in the placenta after adjusting for certain covariates, and only one CpG site remained significant with Bonferroni correction for multiple. In line with the aforementioned prior studies, we also found that PTB was associated with DNA methylation of CpG sites on *PNPLA3, UCN, SLC44A2* and *SLC30A3* in the placenta after adjusting for the covariates, but these associations were not significant after further correcting for multiple testing.

Regarding the association between PTB and DNA methylation in cord blood, a more extensive range of genome-wide studies have been reported. For instance, using the primary high-throughput technique (namely, the Illumina HM27 BeadChip), Schroeder et al. identified that DNA methylations in 39 genes covering 41 CpG sites in cord blood were associated with gestational age, including *AVP*, *OXTR*, *CRHBP* and *ESR1* [28]. Afterwards, using the well-acknowledged Illumina HM450 K BeadChip, studies by Fernando et al., Cruickshank et al., Parets et al. respectively found that PTB was associated with the aberrant DNA methylation in a series of genes involved in the following biological functions including fetal development [29, 31, 52], neurogenesis [31], myometrial relaxation and contraction [29], and DNA methylation regulation [30], but the study of Hong et al. found no significant PTB-associated DNA methylation alteration in cord blood [32]. Furthermore, there were also several studies that assessed the associations between PTB and DNA methylation of specific genes in cord blood, for instance, Burris’s studies discovered the associations of gestational age with *LINE-1* [53] and *AHRR* [54], while Kantake et al. [55] reported altered methylation of *GR* in preterm infants. With the EPIC BeadChip, the current study was able to discover 21 novel DMPs in cord blood that were not covered by the 450 K BeadChip, 14 of them were annotated to specific genes. Another 10 sPTB-associated CpG sites in the cord blood were covered by the 450 K BeadChip, six of them (cg18598117 and cg02001279 on *ARID3A*, cg11932158 and cg18623216 on *PLCH1*, cg04347477 on *NCOR2*, and cg08943494 on *PRR5L*) replicated results from the previous study [31]. Moreover, in accordance with several studies aforementioned, we also discovered that sPTB was significantly associated with altered DNA methylation of several other genes in cord blood, including *ITGB2, RAPGEF3,* and *IGF2BP1*. Besides, we replicated the findings from previous studies [23, 28, 29] which indicated that PTB was associated with DNA methylation changes of *AVP*, *OXTR*, *CRHBP, OTOF, MYH7B, GSK3B, and DNMT1* in cord blood, while these associations were insignificant after further correcting for multiple testing. On the contrary, we did not observe aberrant DNA methylation changes in *MMPs, LINE-1,* or *GR*, which were found to be associated with PTB or gestational age in previous studies. Additionally, we identified two significant CpG sites (cg23039807 and cg23208717) with small β-value differences between PTB and control. Although these two CpG sites remained significant in the sensitivity analysis (Additional file 1: Figure S1), and the regression models were generally robust (Additional file 2: Figure S2), potential model inflation and the risk of false positive could not be completely ruled out. Thus, further validation of these CpG sites are needed to confirm their associations with sPTB and biological significances.

Concerning the inconsistent findings between our study and these previous studies, plausible reasons might be explained as follows. First, all prior studies using the 450 K BeadChip were conducted among Caucasian or African American populations, leaving the Asian population undiscussed, and probes that could be influenced by genetic variants were processed with different approaches. For instance, Fernando et al. did not mask probes targeting CpG sites that covered SNPs, Parets et al. chose to separate these CpG sites for further meQTLs analysis, and the other studies masked only probes targeting CpG sites that covered SNPs but not probes with SNP affecting the extension base. Thus, genetic variants should be taken into account when interpreting results from different ethnicity. The current study was carried out in a Chinese population and we excluded probes targeting CpG sites that may be influenced by SNPs with East Asian minor allele frequency (MAF) over 1% [39]. Four cord blood CpG sites identified in this study were not reported in prior studies, they all overlapped with one or more SNPs with differed MAF among ethnicities [56], and cg26690511 overlapped with rs78091351 which had over 1% MAF in African. Although the MAFs of these SNPs were low (less than 1%) in East Asian population (Additional file 3: Table S1), we could not rule out the possibility that these genetic variants might lead to inconsistent findings with previous studies. Second, the discrepancy across studies might be caused by different population investigated, as they might encounter distinct risk factors that can alter DNA methylation differently [57]. Third, the different DNA methylation measurement techniques used in many previous studies (i.e., the bisulfite sequencing analysis, Sequenom EpiTYPER or Illumina HM27 BeadChip) were quite different from the Illumina EPIC BeadChip used in our study regarding their coverage and detection sensitivity [58–60]. Fourth, different statistical analysis methods were applied in different studies. For example, Kim’s study prioritized differentially methylated CpG sites by difference score (corresponding to *p*-value of < 0.0001), while we adjusted for covariates and performed Bonferroni procedure to correct for multiple testing, which is a more strict approach with lower false discovery rate than the former, and thus was reasonable to yield less significant DMPs. Fifth, unlike other studies (e.g., Burris’s studies [53, 54]) which had a much larger sample size and thus more power to detect the difference of DNA methylation level between PTB and term birth, ours’ employed a relatively small sample size. Sixth, many previous studies focused on gestational age as the outcome variable while we only examined sPTB. Although these two outcomes shared some similar implications, they were inherently different and might lead to statistically and clinically inconsistent results.

Taken together, despite certain inconsistencies, findings of the current study provide vital implication that DNA methylation in placenta and cord blood may be associated with sPTB.

### Comparison of sPTB-associated DNA methylation variations in placenta and cord blood

Distinct level of DNA methylation was observed in sPTB-associated CpG sites between placenta and cord blood in this study, and more interestingly, the genes covering sPTB-associated CpG sites in placenta and cord blood showed different tendencies towards the enriched biological processes. More of the genes in placenta were involved in biological regulation and response to stimulus, while higher proportions of those in cord blood were related to metabolic process. These differences may be attributed to tissue specificity of DNA methylation [61], and may also imply that placenta and fetus may respond differently during the occurrence of risk factor-induced PTB.

Accumulating evidence has shown that maternal environmental risk factors during pregnancy may impair the placenta directly [62, 63] or cause DNA methylation alterations which further results in the permanent structural and functional changes of the placenta [64–66]. For example, the genes encoding vascular endothelial growth factor (VEGF) and its receptor (Kinase insert domain receptor, KDR) were found to have altered DNA methylation status in the PTB placenta [27]. DNA methylation alternation may change the expression of these two angiogenic factors, which would affect the development and structure of placental vascular and thereby leading to PTB [67–69]. Similarly, our study also found altered methylation of *VEGF* and *KDR* in placenta (although not significant after multiple testing correction). Therefore, maternal exposure to environmental factors may alter placental DNA methylation of genes that are mainly involved in maintaining the structure and function of the placenta.

On the over hand, if the structure or function of the placenta is impaired by hazardous environmental factors, the placenta would be incapable of transporting necessary nutrients to the fetus or protecting it from detrimental substances [11, 70]. Consequently, the fetus would have to suffer from an impaired nutritional environment, decreased energy metabolism and altered metabolic programming [71, 72]. These might account for the finding in our study that genes with DNA methylation alternation in sPTB cord blood were principally enriched on metabolic process. In line with our study, many previous studies also identified DNA methylation changes of genes related to metabolism in PTB cord blood [29, 30, 52], such as *NCOR2*, *IGF2BP1*, *IGF2,* and *TET1*. Additionally, we also observed DNA methylation alterations of *BAIAP2* and *OXTR* in sPTB cord blood, which were related to neurodevelopmental disorders. To specify, *BAIAP2* encodes brain-specific angiogenesis inhibitor - binding protein that is involved in neurodevelopmental/ neurite outgrowth network [73], and its genotype and expression were associated with autism spectrum disorders (ASD) [74] and attention-deficit/hyperactivity disorder (ADHD) [73, 75, 76]. The methylation of the oxytocin receptor (*OXTR*) was also associated with ASD [25, 77].

However, it is still unresolved regarding whether these DNA methylation variations in placenta and cord blood are potential molecular mechanisms in the association of sPTB with subsequent long-term metabolic and neural developmental disorders, and further birth cohort studies are warranted to assess their relationships.

### Strengths and limitations

The current study is the first genome-wide assessment on the association of DNA methylation and spontaneous preterm birth in the Chinese population using the Illumina EPIC BeadChip, which enabled us to detect several novel sPTB-associated CpG sites that were not covered by the previous 450 K BeadChip. Moreover, we simultaneously examined DNA methylation of placenta and cord blood samples, so that both placental and fetal factors could be taken into account in the process of sPTB.

On the other hand, a few limitations of our study should be addressed. First, owing to the relatively small sample size, our study might not have enough power to detect some significant DMPs between sPTB and term birth. Second, we neither used validation samples nor conducted additional examinations to validate the high-throughput data; our data were only compared and validated to previous studies. Third, the current study could not assess the expression of the identified sPTB-associated genes with transcriptome data and confirm the influence of DNA methylation alteration. Fourth, due to the cell specificity of DNA methylation, an ideal study design would be to examine DNA methylation in each cell type separately. However, our study used whole cord blood, a mixed-cell sample in which the overall DNA methylation levels could be influenced by cell composition [78, 79]. As a result, the DMPs that we identified may not be attributed to differential methylation in specific cell lines, although cell composition proportion was controlled. Fifth, causal relationship between DNA methylation changes and sPTB could not be determined, as the biological samples were collected at delivering.

## Conclusion

DNA methylation variations in placenta and cord blood were associated with spontaneous preterm birth, yet the genes with DNA methylation variations appeared to show different patterns of biological process enrichment between the two tissues. These findings can bring novel insights into how DNA methylation patterns may associate with spontaneous preterm birth and its health consequences.

## Additional files

Additional file 1: **Figure S1.** Sensitivity analysis of placenta and cord blood models. Scatter plots comparing negative log_10_*p*-values between the main analysis models [placenta (A) and cord blood (B) DNA methylation with respect to sPTB status, adjusted for maternal age, newborn gender, maternal education level, pre-pregnancy BMI, and when appropriate, either the first five principal components obtained from the ReFACTor function or estimated cell-type proportions] on the horizontal axis of A-B, and sensitivity analysis models [placenta (A) and cord blood (B) DNA methylation with respect to sPTB status, adjusted for either the first five principal components obtained from the ReFACTor function or estimated cell-type proportions when appropriate] on the vertical axis of A-B. All the significant CpG sites in cord blood remained significant (Bonferroni corrected *p* < 0.05) in the sensitivity analysis models, the one significant site in placenta did not pass Bonferroni correction but was still the top site among all. The diagonal line across the two scatterplots represents y = x. (TIF 376 kb)

Additional file 2: **Figure S2.** Q-Q plots and *p*-value distribution histograms of placenta and cord blood models. A and B. The quantile-quantile (Q-Q) plots comparing observed probability distribution against expected distribution. The shading indicates the 95% confidence intervals. The inflation factors, λ were 1.266 in placenta model (A) and 1.477 in cord blood model (B). C and D. The histograms showing the distributions of *p*-values of placenta (C) and cord blood (D) regression models. (TIF 247 kb)

Additional file 3: **Table S1.** Overlapped SNPs of the sPTB-associated DMPs covered by the 450 K BeadChip. Overlapped known SNPs of the sPTB-associated DMPs covered by the 450 K BeadChip, with minor allele frequencies among East Asian, European (Caucasian), and African populations. (XLSX 11 kb)

## Abbreviations

ADHD: Attention-deficit/hyperactivity disorder
ASD: Autism spectrum disorders
DMP: Differentially methylated position
FT-NBW: Full-term birth with normal birthweight
GA: Gestational age
GDM: Gestational diabetes mellitus
GO: Gene Ontology
HDP: Hypertensive disorders during pregnancy
KDR: Kinase insert domain receptor
LMP: Last menstrual period
MAF: Minor allele frequency
OXTR: Oxytocin receptor
PPROM: Premature rupture of membranes
PTB: Preterm birth
sPTB: Spontaneous preterm birth
VEGF: Vascular endothelial growth factor
