# Supplementary figures and images for: Comparison of DNA methylation profiles associated with spontaneous preterm birth in placenta and cord blood
Source: BMC Med Genomics. 2019 Jan 3;12:1. doi: 10.1186/s12920-018-0466-3 (PMC6318854; doi:10.1186/s12920-018-0466-3)

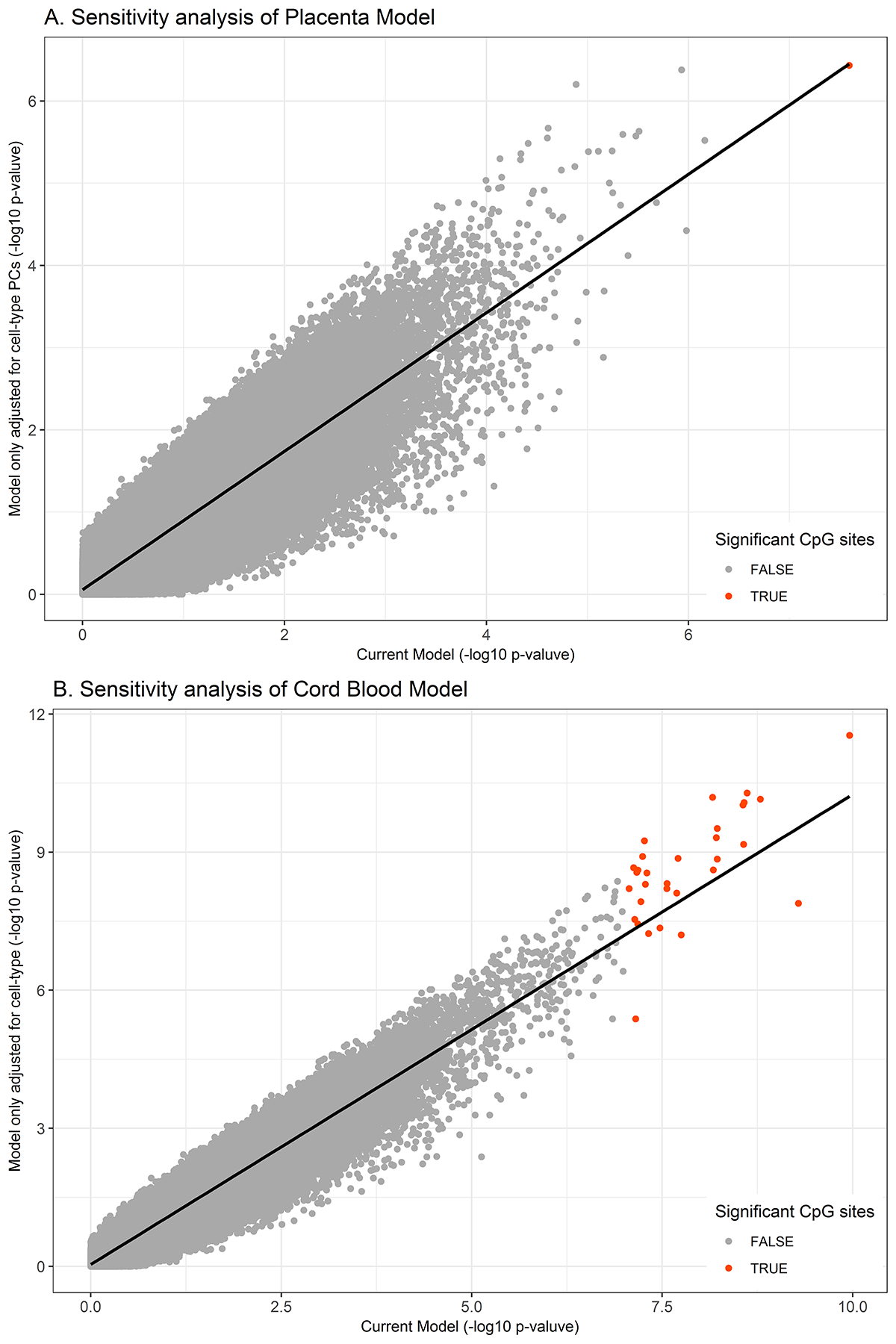

Supplement: Supplementary file 1 — Figure S1. Sensitivity analysis of placenta and cord blood models. Scatter plots comparing negative log10p-values between the main analysis models [placenta (A) and cord blood (B) DNA methylation with respect to sPTB status, adjusted for maternal age, newborn gender, maternal education level, pre-pregnancy BMI, and when appropriate, either the first five principal components obtained from the ReFACTor function or estimated cell-type proportions] on the horizontal axis of A-B, and sensitivity analysis models [placenta (A) and cord blood (B) DNA methylation with respect to sPTB status, adjusted for either the first five principal components obtained from the ReFACTor function or estimated cell-type proportions when appropriate] on the vertical axis of A-B. All the significant CpG sites in cord blood remained significant (Bonferroni corrected p < 0.05) in the sensitivity analysis models, the one significant site in placenta did not pass Bonferroni correction but was still the top site among all. The diagonal line across the two scatterplots represents y = x. (TIF 376 kb) [file 12920_2018_466_MOESM1_ESM.tif]

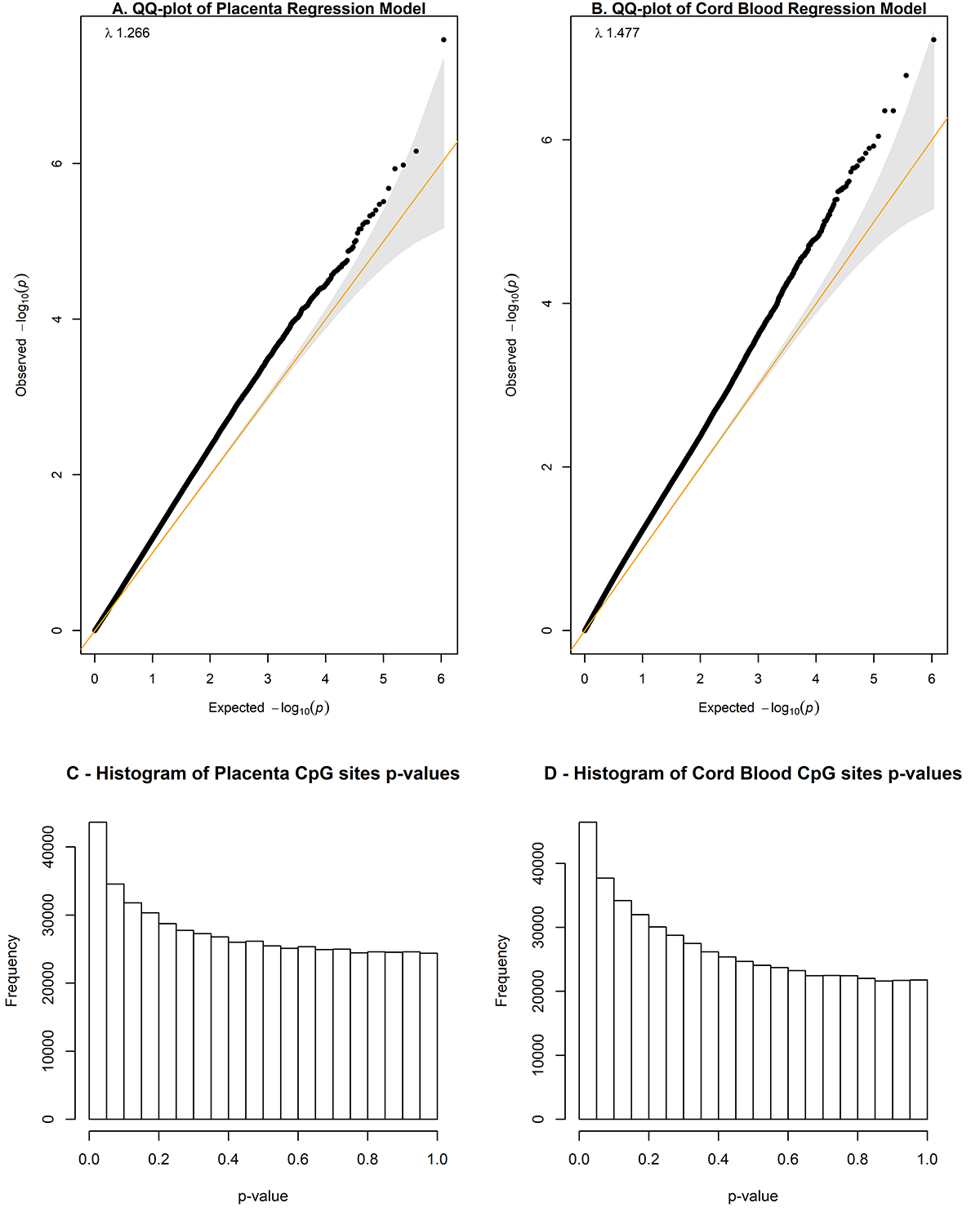

Supplement: Supplementary file 2 — Figure S2. Q-Q plots and p-value distribution histograms of placenta and cord blood models. A and B. The quantile-quantile (Q-Q) plots comparing observed probability distribution against expected distribution. The shading indicates the 95% confidence intervals. The inflation factors, λ were 1.266 in placenta model (A) and 1.477 in cord blood model (B). C and D. The histograms showing the distributions of p-values of placenta (C) and cord blood (D) regression models. (TIF 247 kb) [file 12920_2018_466_MOESM2_ESM.tif]
